# Effect of Different Forms of Antimony (Sb) on Genes Encoding Functions Associated with Root Morphology, Physiology and Root Cell Wall in Rice (*Oryza sativa*)

**DOI:** 10.3390/plants15152297

**Published:** 2026-07-27

**Authors:** Syed Muhammad Azam, Yang Liu, Jiaxin Dai, Ziting Lin, Li Yang, Shengjie Shi, Jigang Yang, Pingping Zhao, Yanshuang Yu, Zhilian Fan, Hend Alwathnani, Madeha A. Alonazi, Christopher Rensing, Hong Liu, Shunan Zheng, Renwei Feng

**Affiliations:** 1 Institute of Environmental Microbiology, College of Resources and Environment, Fujian Agriculture & Forestry University, Fuzhou 350002, China; syedazamfafu@gmail.com (S.M.A.); dxj_lg@163.com (J.D.); linziting@126.com (Z.L.); yanglicolor@163.com (L.Y.); yangigxj@foxmail.com (J.Y.); zpingping@163.com (P.Z.); yuyanshuang2018@163.com (Y.Y.); fjauliuhong@163.com (H.L.); 2 Institute of Agro-Environmental Protection, Ministry of Agriculture, Tianjin 300191, China; 17797995009@163.com; 3 Agricultural College, Guangxi University, Nanning 530004, China; 18338648393@163.com (S.S.); fanzhilianl@163.com (Z.F.); 4 Department of Botany and Microbiology, King Saud University, Riyadh 11451, Saudi Arabia; wathnani@ksu.edu.sa (H.A.); malonze@ksu.edu.sa (M.A.A.); 5 Rural Energy and Environment Agency, Ministry of Agriculture and Rural Affairs, Beijing 100125, China; zhengshunan1234@163.com

**Keywords:** antimony, root morphology, plant physiology, heavy metal toxicity, enzymatic activity

## Abstract

High levels of antimony (Sb) adversely affect plant growth and development. We aimed to uncover the harmful effects of different forms of Sb on root morphology, physiology and expression profiles of genes encoding functions associated with roots. Rice plants grown in ½ Hoagland nutrient solution were exposed to Sb(III) and Sb(V) at concentrations of 10 and 20 mgL^−1^ for one week. Results demonstrated that higher concentrations of Sb(III) significantly impaired root morphological traits, with high toxicity observed at 20 mgL^−1^ Sb(III) and 10 mgL^−1^ Sb(V). The application of Sb(III) led to reduced uronic acid levels in hemicellulose-II (HCII) with cell organelle and cytosol displaying substantial accumulation of Sb(III). Enzymatic activity revealed that high levels of Sb(III) disrupted the activity of cellulase (CE) and pectin methylesterase (PME), while augmenting the activity of Xyloglucan endotransglycosylase hydrolase (XTH). Additionally, polygalacturonase (PG) was significantly reduced under Sb(V) 10 mgL^−1^ exposure. Pearson’s correlation coefficient was used for continuous data to draw a linear trend between studied parameters. Shoot biomass displayed a negative correlation with root and shoot Sb. Shoot XTH had a positive correlation with shoot Sb. Furthermore, root and shoot Sb showed a positive association with root diameter and XTH. Hemicellulose-1 (HCI) was negatively associated with PME, PG and pectin, suggesting that HCI was a suitable binding site for uronic acid in the context of Sb contamination. Additionally, to elucidate the molecular mechanisms underlying root structural alterations, the expression profiles of key cell wall-related genes—*Expansin*, *Cellulase synthase*, *XTH8* (*xyloglucan endotransglucosylase/hydrolases*) and *Pectinesterase*—were systematically analyzed using qRT-PCR. All genes were up-regulated in response to different forms of Sb exhibiting resistance. *Xylanase* was down-regulated, showing its role in the containment of Sb in roots. Further studies are advised for elucidating the mechanism of action of Sb on different tissues of rice plants.

## 1. Introduction

Antimony (Sb) is a member of Group V (metalloid). As earlier reports have pointed out, global antimony mining reached 110,000 tons in 2022, with China being the largest producer (55%) of the global output [1]. Sb was historically overlooked compared to other heavy metals and metalloids; however, the global consumption of Sb has accelerated dramatically in recent years. Sb is primarily used in advanced automotive sectors, where it serves as a critical component in lead batteries, microelectronics, semiconductors and diodes, as well as a key synergistic flame retardant in automotive plastics and textiles [2]. This surge in manufacturers’ demands has increased mining and thus resulted in Sb massive anthropogenic releases into surrounding ecosystems, thus contaminating soil, specifically water.

Antimony exists in several oxidation states, e.g., −3, 0, +3 and +5, and has a very strong affinity for clay [3], with antimonite Sb(III) and antimonate Sb(V) being predominant in soils [4]. A previous study determined that Sb3+ is ten times more hazardous than Sb5+. Due to its possible toxicity and carcinogenicity, the US Environmental Protection Agency (US EPA) has categorized Sb as a priority pollutant. Similarly, the EU Basel Convention has also listed it as hazardous waste [5]. China is leading the world in mining and reserves of Sb; moreover, at this point, it holds >90% of the world’s Sb production, followed by Australia and Russia. In the Hunan province of China alone, the concentration of Sb is at 5045 mg/kg [6]. Due to its tenacious nature and high mobility in terrestrial and aquatic matrices, Sb readily enters the food chain via agricultural soils, posing a direct threat to human and animal health through dietary intake. Sb is non-essential to plants but can still obtain access to enter the plants roots through the aquaglyceroporin transport channel, specifically the nodulin intrinsic proteins (NIPs), where it is readily absorbed by plants and is able to cause plant death under high concentrations [7]. In Sb-contaminated soils for grazing, pasture plant species and crops have been reported to accumulate precarious concentrations of Sb [8]. Both above ground and underground surfaces of plants often receive heavy metal(loid)s stress through elevated exposure. Furthermore, the cell wall components, pectin and hemicellulose, have been shown to play a critical role in the binding of heavy metal(loid)s, hindering the uptake of essential nutrients by plants [3,6,9]. The plant root is considered an important entrance for the absorption of heavy metal ions by plants [10]. Plants are able to adapt to negative stressors, such as lead stress [11] and Sb(III, V) stress [7,12]. Rice root morphological characteristics have been found to be significantly reduced by high levels of Sb(III) [7,13]. However, Sb(V) was found not to affect the above parameters but instead to reduce the root biomass [7], suggesting that there are differences in toxicity between Sb(III) and Sb(V) with regard to plant roots. Root development is associated with cell growth parameters [14].

The cell wall has shown to function as a barrier or a reservoir for heavy metals in plants [15,16] via the binding of metallic cations with negatively charged functional groups [7,17]. Similarly, a large amount of Sb was found to be sequestrated in the cell wall [7,12,18].

The plant primary cell wall is mainly composed of polysaccharides (90–95%) and proteins (5–10%), while the lignified secondary wall is composed of polysaccharides (about 65%) and lignin (about 35%) [19]. Polysaccharides mainly consist of cellulose, hemicellulose and pectin [10,16,20]. Cellulose enables the maintenance and structural integrity of cells and the cell wall [21], and pectin plays a crucial role in cell adhesion [22], cell expansion and elongation [23,24], cell wall strength and porosity [23,25,26], cell growth, cell differentiation, determining the integrity and rigidity of plant tissue [23]. This marks pectin as an important factor in plant root development. Sb(III) and Sb(V) have both been able to significantly increase the contents of lignin, pectin and calcium [7], suggesting that they have roles in sequestrating Sb or resisting Sb stress in plants.

PG and PME take part in the modification of pectin [27,28]. Pectins have been shown to be directly secreted into the cell wall [10,28]. In addition, PME plays an auxiliary role in cellular separation [29], seed germination [18], root tip elongation [30], and leaf growth polarity [31]. The enzyme β-galactosidase (β-gal) is able to remove specific sugar residues from hemicellulose and pectin from the cell wall [32]. XTHs helps in the remodeling of cellulose/xyloglucan crosslinks to regulate cell wall extension, cell elongation, and stress resistance [33,34], which are considered stress adaptations [35,36] and cell morphogenetic processes [34]. The root cell wall is the first contact site for Sb uptake and detoxification, and the structural and functional integrity largely depends on the integrated action of cell wall-associated genes encoding functions, such as *XTHs* (*xyloglucan endotransglucosylase/hydrolases*), *expansins*, *pectinesterase* and *cellulose synthase* [37,38]. Despite the information linking these genes to abiotic stress tolerance, their peculiar roles in regulating rice root growth and cell wall integrity as well as remodeling under antimony stress have not been investigated, requiring detailed expression and functional analyses of root-cell-wall-related genes [39].

Since Sb(III) and Sb(V) can affect the root morphology and be stored in the cell wall, as well as being able to enhance the contents of pectin and lignin at different doses, we propose the following hypothesis: Sb can affect plant root morphology by changing enzyme activities and thus the synthesis of root cell wall components, ultimately affecting element absorption and distribution. Therefore, this study was designed with rice variety (Yexiangyou 3) using hydroponic experiments to study the effects of Sb(III) and Sb(V) on the following processes: (1) the activities of hydrolase enzymes in the cell wall and their correlation with the synthesis of cell wall components, and thus with root morphological changes; (2) the roles of the root cell wall in binding Sb (3); and the expression profiles of genes encoding root-cell-wall-related functions under exposure to different speciation of Sb. Furthermore, an analysis of *cis*-regulatory elements of the promoter sequences of genes encoding root-cell-wall-related functions was performed.

## 2. Results

The objectives of this study were to study the effects of different forms of Sb on root morpho-physiological characters, cell wall components, and correlated enzymes of the rice plant root. Generally, toxicity of Sb(III) was higher than that of Sb(V), while Sb(III)’s toxicity was higher in roots. Sb’s toxicity was high in the cell wall and cytosol. β-gal was affected at a low concentration of Sb(III), while PME was affected at a high concentration. Sb increased uronic acid in HCI and HCII.

### 2.1. Sb Uptake and Its Effect on Root and Shoot Biomass

**Figure 1:**
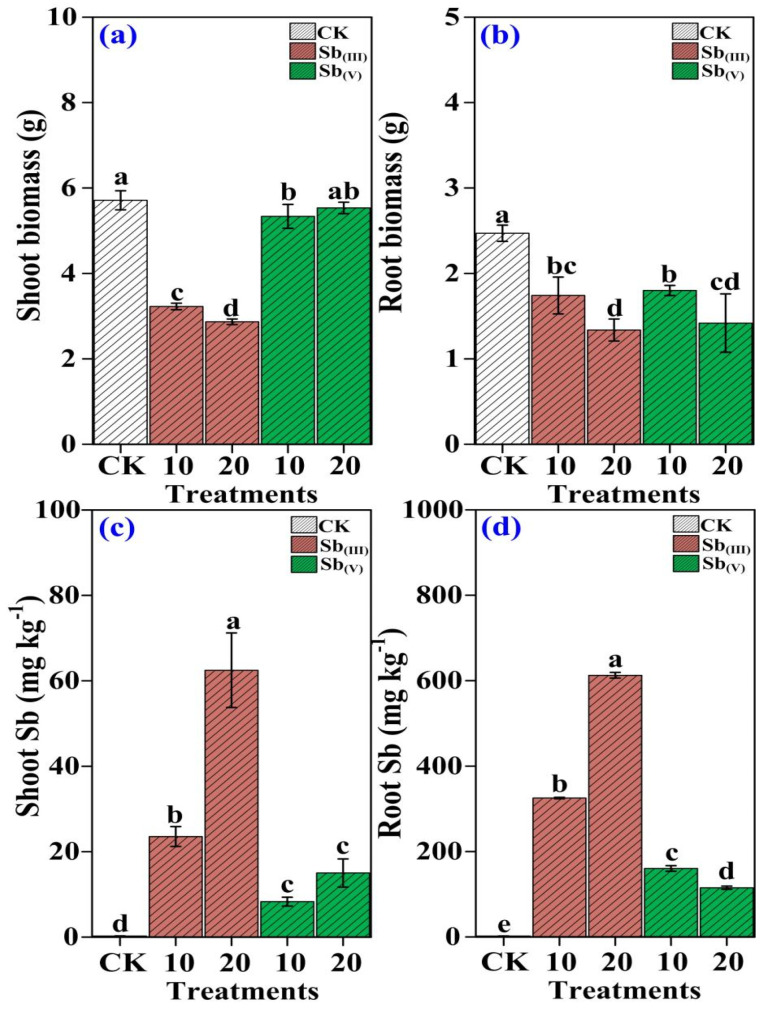
Biomass responses and tissue accumulation of antimony in plants. Graphs show (**a**,**b**) reductions in shoot and root biomass and (**c**,**d**) increased shoot and root Sb concentrations under varying concentrations of Sb(III) and Sb(V) (10 and 20 mg·kg^−1^) compared to the control (CK). Different lowercase letters indicate significant differences at *p* < 0.05.

Various physiological and biochemical processes in plants have been reported to be affected by heavy metal contamination. In the current experiment, different speciation of Sb were applied to rice plants at different concentrations. Higher concentrations of SbIII 20 mg L^−1^ significantly reduced rice plant root and shoot biomass (g) when compared to the control, while Sb(V) 10 and 20 mg L^−1^ only slightly reduced shoot biomass (Figure 1a). Moreover, root biomass was significantly reduced by both forms of Sb (Sb(III), Sb(V)) at both levels (10 and 20 mg L^−1^) compared to the control (Figure 1b). Furthermore, the dried root and shoot samples were analyzed for Sb content (mg L^−1^). Shoot Sb uptake was increased under Sb(III) at both levels. In contrast, Sb(V) uptake was less when compared with Sb(III) at both concentrations (Figure 1c). Results showed that roots were more vulnerable to Sb toxicity. Root tissues displayed a high uptake of Sb(III) at a high concentration of Sb(III) 20 mg L^−1^. A mild change in uptake was observed under different levels of Sb(III) in root tissues (Figure 1d). Sb(V) uptake varied with different concentrations; in rice roots, Sb(V) 10 mg L^−1^ uptake was higher than Sb(V) 20 mg L^−1^ uptake, while a significant change was observed in shoot Sb(V) accumulation. Briefly, rice roots accumulated significant amounts of Sb(III) and Sb(V) while sustaining less biomass damage under pentavalent Sb(V) exposure. Therefore, selecting and modifying root-anchored traits could offer a viable strategy for the phytostabilization of antimony-polluted soils.

### 2.2. Galacturonic Acid and Total Sugar Concentrations of Pectin, HCI and HCII

**Figure 2:**
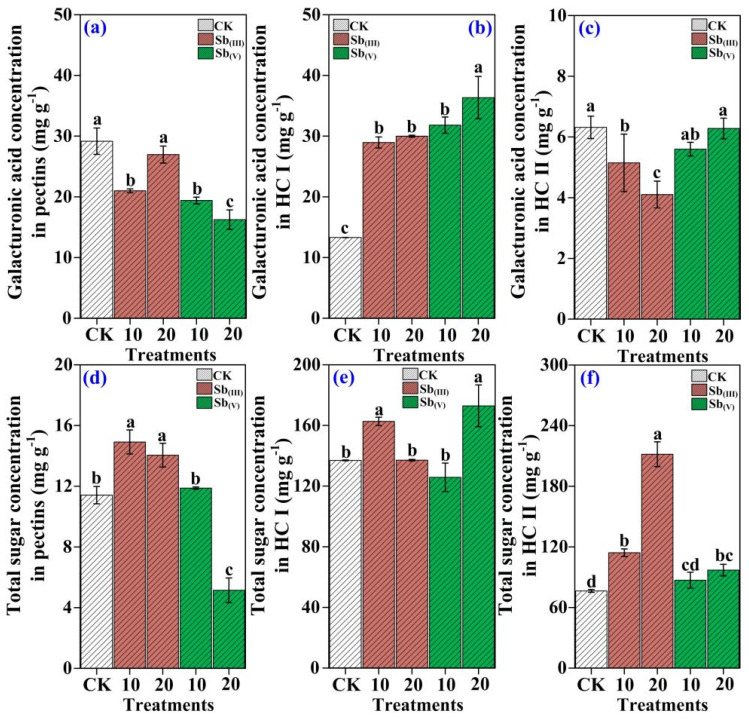
Galacturonic acid concentrations in (**a**) pectin, (**b**) HCI, and (**c**) HCII in response to Sb (Sb(III) and Sb(V) at 10 and 20 mg L^−1^). Total sugar concentration (mg·g^−1^) alteration in (**d**) pectin, (**e**) HCI, and (**f**) HCII. The different lowercase letters above bars indicate significant differences (*p* ≤ 0.05).

Uronic acid concentrations in different cell wall components were measured in control plants. Pectin was shown to have high uronic acid concentrations, followed by HCI and HCII. However, when rice plants were treated with Sb, results revealed that uronic acid content in pectin and HCII was less than that in the control for Sb(III) and Sb(V) at both lower and higher concentrations, except for Sb(V) at 20 mg·kg^−1^ (Figure 2a,c). However, Sb(III) and Sb(V) at higher and lower concentrations resulted in an increased content of uronic acid in HCI (Figure 2b).

Furthermore, we observed the total sugar concentration in rice in response to Sb application. An increase in total sugar concentration was observed in Pectins under Sb(III) at 10 and 20 mg L^−1^. Sb(V) did affect sugar contents under 10 mg L^−1^, and a significant decrease was noted in sugar contents under Sb(V) 20 mg L^−1^ (Figure 2d). Sb(III) 10 mg L^−1^ and Sb(V) 10 mg L^−1^ had significantly increased sugar contents in HCI (Figure 2e). A significant increase in total sugar content was noticed under Sb(III) at 10 mg L^−1^ and 20 mg L^−1^ and under Sb(V) at 10 and 20 mg L^−1^ in HCII. Sugar content was significantly higher in HCII under Sb(III) 20 mg L^−1^ (Figure 2f).

### 2.3. Concentration of Sb (%) in Different Cell Wall Components

**Figure 3:**
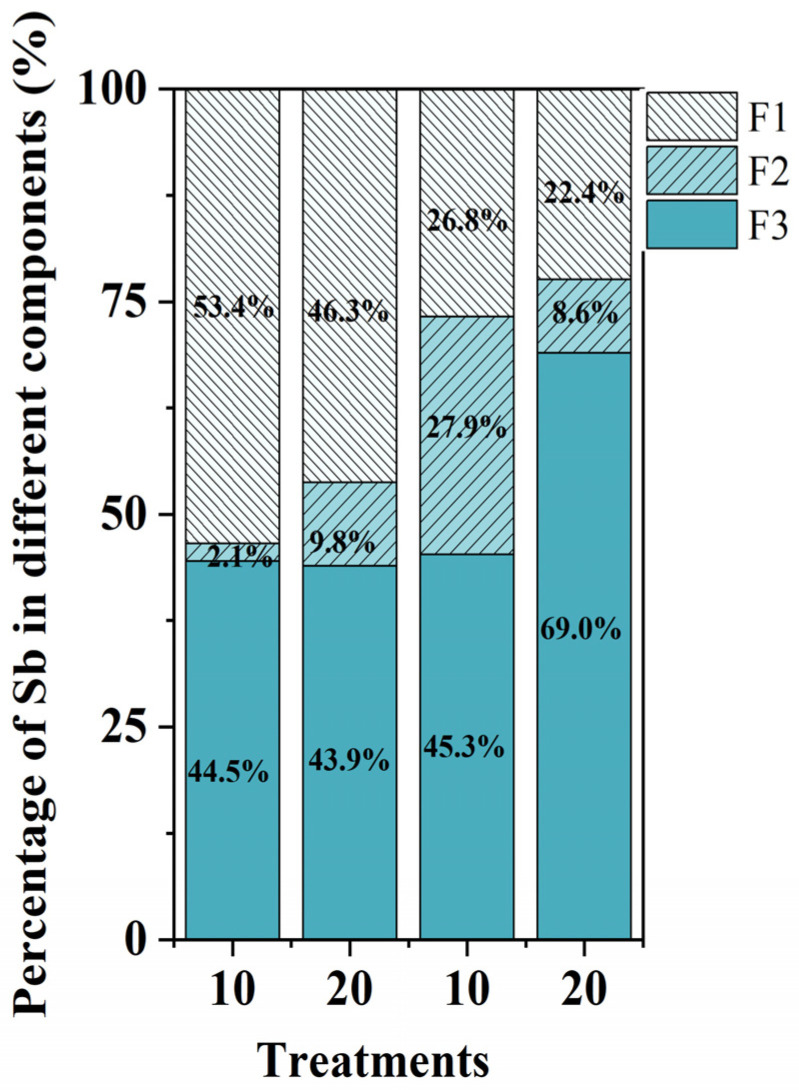
Concentration of Sb (%) in different cell wall components: F1 (cell wall), F2 (organelle component), and F3 (cytosol). Rice plants were treated with Sb (Sb(III) at 10 and 20 mg L^−1^ as well as Sb(V) at 10 and 20 mg L^−1^) for one week. Data is presented in each compartment as a percentage. The treatments were considered to be significantly different at *p* ≤ 0.05.

Rice roots sub-cellular components, e.g., F1 (cell wall), F2 (organelle component), and F3 (cytosol), were isolated and screened for Sb accumulation (Figure 3). F1 contained 53.4% and 46.3% Sb(III) 10 mg L^−1^ as well as Sb(III) 20 mg L^−1^, respectively. F2 contained 2.1% Sb(III) 10 mg L^−1^ and 9.8% Sb(III) 20 mg L^−1^. F3 did not show significant variations in Sb accumulation at both levels. In the case of Sb(V), F1 contained 26.8% and 22.4% at Sb(V) 10 mg L^−1^ and Sb(V) 20 mg L^−1^, respectively. F2 had a significantly high content (27.9%) of Sb(V) at a low concentration, while Sb(V) accumulation decreased (8.6%) at a higher concentration. F3 contained less Sb(V) (45.3%) at Sb(V) 10 mg L^−1^ but contained a high amount of Sb(V) at a higher level (69%) (Figure 3).

### 2.4. Cell Wall-Related Enzyme Activities in Rice Roots

**Figure 4:**
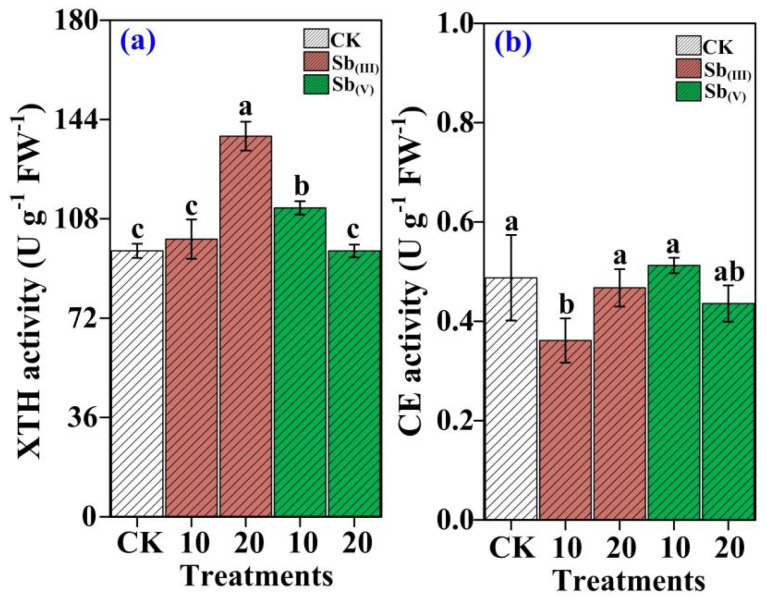
Activities of different cell wall enzymes, including XTH (**a**) and CE (**b**), in rice roots under Sb(III) at 10 and 20 mg L^−1^, Sb(V) at 10 and 20 mg L^−1^ stress. The different lowercase letters above bars indicate significant differences between treatments for different enzymes (*p* ≤ 0.05).

Different cell wall enzymes activities were investigated for their responses to different forms of Sb. The rice cell wall enzyme XTH showed to have an activity that was significantly increased under Sb(III) 20 mg L^−1^ and Sb(V) 10 mg L^−1^, while a non-significant effect was observed under Sb(III) 10 mg L^−1^ and Sb(V) 20 mg L^−1^ (Figure 4a). CE’s activity was non-significant under Sb(III) 20 mg L^−1^, Sb(V) 10 mg L^−1^ and Sb(V) 20 mg L^−1^, while CE’s activity was significantly reduced under Sb(III) 10 mg L^−1^ (Figure 4b).

**Figure 5:**
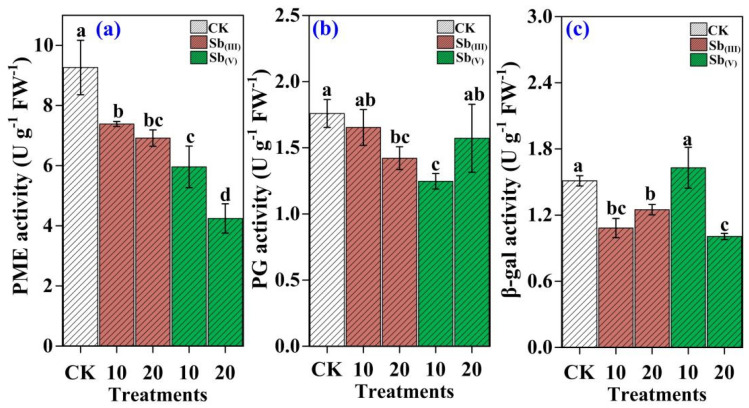
Activities of different cell wall enzymes, including PME (**a**), PG (**b**), and β-gal (**c**) in rice roots of rice plants under Sb(III) at 10 and 20 mg L^−1^ and Sb(V) at 10 and 20 mg L^−1^. The different lowercase letters above bars indicate significant differences between treatments for different enzymes (*p* ≤ 0.05).

Furthermore, the PME and PG activities were significantly reduced under Sb(III) at 10 and 20 mg L^−1^, Sb(V) at 10 and 20 mg L^−1^ stress. PME activity was reduced under Sb(V) at both levels compared to Sb(III) (Figure 5a). PG’s activity was slightly reduced under Sb(III) 10 mg L^−1^ and Sb(V) 20 mg L^−1^, while PG’s activity significantly decreased under Sb(V) 10 mg L^−1^ (Figure 5b). β-gal activity in rice root cell was non-significantly affected by Sb(V) 10 mg L^−1^, while β-gal activity was significantly reduced under Sb(III) 10 mg L^−1^, Sb(III) 20 mg L^−1^ and Sb(V) 20 mg L^−1^ (Figure 5c).

### 2.5. Effects of Different Forms of Sb on Phenotypic Traits of Roots

Rice root morphology was affected by different forms of Sb. Different root morphological parameters were measured (Supplementary Table S1). Compared to the control, a slight increase was observed in root length at lower levels of Sb(III) and Sb(V), while a significant decline in root length was observed in response to higher levels of Sb(III) and Sb(V). The root surface area was reduced at high levels of both forms of Sb. The root volume was significantly decreased after exposure to Sb(III) 20 mg L^−1^, followed by Sb(V) 10. In contrast, the root diameter was increased in rice plants treated with Sb(III) and Sb(V) at both levels. Sb(III) and Sb(V) reduced the number of tips and root length with an increasing concentration as compared to the lower concentrations. Collectively, our results demonstrate that high concentrations of Sb, particularly Sb(III), strongly disturbed the structural architecture of the rice root system by inhibiting longitudinal growth and surface expansion while inducing a compensatory increase in root thickness.

### 2.6. Correlation Analysis Between Qualitative and Quantitative Traits in Rice Under Sb Stress

Pearson’s correlation was utilized for qualitative and quantitative traits, and root-cell-wall-related enzymes were analyzed for their interdependence after plants’ exposure to different forms of Sb. Phenotypic correlation was broadly categorized as a positive and negative correlation, although statistics were applied, showing highly significant and significant correlations (** *p* < 0.01 and * *p* < 0.05, respectively) (Supplementary Table S2).

#### 2.6.1. Biomass- and Tissue-Specific Sb Content

Root and shoot biomass were significantly and negatively correlated with root and shoot Sb content, indicating the negative impact of Sb on the vegetative growth of the plant. Shoot and root biomass showed to have no association, while root and shoot Sb content had a positive correlation.

#### 2.6.2. Tissue-Specific Sb and Cell Wall Enzymes

Root and shoot Sb had a significantly positive correlation with XTH only, while PG, β-gal, CE, and PME had a non-significant correlation with tissue-specific Sb.

#### 2.6.3. Tissue-Specific Sb and Cell Wall Components

Root and shoot Sb displayed a significant negative correlation with HCII, while other cell wall components did not have significant correlation with root and shoot Sb.

#### 2.6.4. Tissue-Specific Sb and Growth Parameters

Growth parameters, like leaf area, number of tips, volume, and root length, were negatively correlated with root and shoot Sb. Root diameter was positively correlated with root and shoot Sb.

### 2.7. Expression Profiles of Root-Cell-Wall-Encoding Genes and Cis-Regulatory Elements

**Figure 6:**
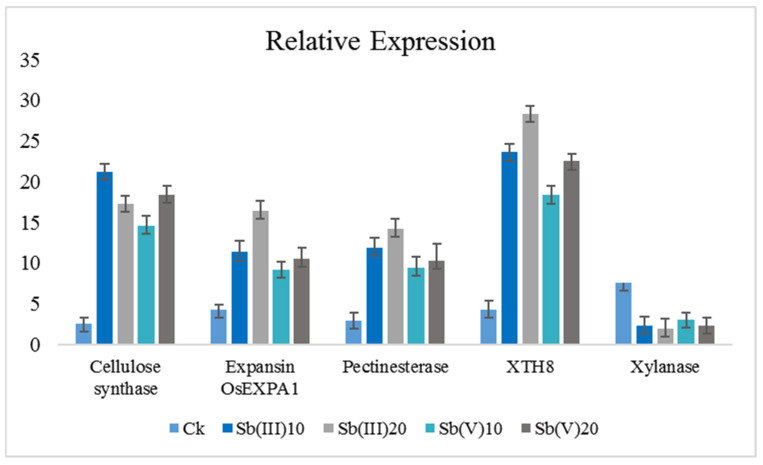
Expression profiles of genes encoding root-associated functions under different forms of Sb (Sb(III) at 10 and 20 mg L^−1^, Sb(V) at 10 and 20 mg L^−1^) with different concentrations in rice. Expression profiles values are represented the mean data of three replicates from qRT-PCR. Data are presented as mean relative expression values, with error bar representing the standard error of the mean (SEM).

Genes encoding functions associated with rice roots were selected based on their identified functional roles in root cell wall integrity, remodeling, and expansion. Genes, namely *cellulose synthase*, *expansin OsEXPA1*, *pectinesterase*, *XTH8*, and *Xylanase*, were observed in rice under different speciation of Sb (Sb(III) at 10 and 20 mg L^−1^ and Sb(V) at 10 and 20 mg L^−1^). The primers used in qRT-PCR are outlined in Supplementary Table S3. Antimony in two forms was applied to rice plants for one week at 10 mg L^−1^ Sb(III), 20 mg L^−1^ Sb(III), 10 mg L^−1^ Sb(V), and 20 mg L^−1^ Sb(V); there was one control without the application of Sb. The expression profiles were assessed using qRT-PCR, showing that the above-mentioned genes revealed high expression as compared with Ck (Figure 6). Only *Xylanse* was down-regulated in response to both forms of Sb. *XTH8* was highly and significantly expressed compared to other genes.

Furthermore, we assessed the cis-regulatory elements of the above-mentioned root-related genes (Table 1), as cis-acting elements in the gene’s promoter play a critical role in the regulation of gene expression under different biotic and abiotic stimuli. Here, we wanted to explore genes encoding rice root-cell-wall-related functions at the transcription level. The analysis of cis-acting elements of rice root-related genes showed that the promoter sequences containing ABA-responsive elements (ACGTG), dehydration-responsive elements (DREs) (RCCGAC), anaerobic-responsive elements (TGGTTT), and metal-responsive elements (TGCAGGC), which are related to environment stimuli and heavy metals (such as Zn, Cd, and Sb), were associated with plants. There were some elements related to biotic and abiotic stress, such as W-Box (TTGAC-T) and T/G-box (AACGTG), which are pathogen response elements, and the MYB recognition element (CAACTG), which functions as a drought, salt, cold and antioxidant defense element.

## 3. Discussion

Antimony (Sb) is considered one of the of the most toxic elements [40]. Heavy metals such as Cu, Zn, Mo, Co, Ni and Mn are crucial for some biological processes, and some of them are involved in different biological pathways. At the same time, there are highly toxic heavy metal(loid)s, like arsenic (As), mercury (Hg), lead (Pb), cadmium (Cd) and antimony (Sb), which reduce crop productivity through morphological and metabolic disorders when their concentrations exceed optimal levels [41,42]. In the south of China, Guizhou province harbors a Sb mining area, causing water and soil contamination [43]. Heavy metals are in part toxic due to the production of reactive oxygen species (ROS), e.g., superoxide anion radical (O_2_^−^), resulting in the disruption of the redox homeostasis of cells [44]. Some ultra-structural damage caused by Sb in some vegetative parts of *Trapa natans* was also observed using scanning electron microscopy (SEM) and transmission electron microscopy (TEM) [45].

In our current experiment, rice plants were treated with different forms of Sb using different concentrations. Plant root and shoot biomasses were reduced significantly due to higher concentrations of Sb in both forms (Figure 1a,b). Root and shoot Sb had a similar positive correlation with shoot Sb, root diameter, and XTH (Table S2). Earlier studies in maize reported plant growth and biomass reduction with increasing soil Sb concentration [2,6,46]. However, it was also shown that that Sb(V) did not influence root biomass but retarded root growth architecture only [47]. Plants have adopted an effective defense system to endure the toxic effects of Sb; despite this, an excessive concentration of Sb above a certain level defeats the antioxidant defense organization and inhibits plant growth [48]. Regarding the above results, we can say that there is a variety of aspects that is anticipated to influence the Sb uptake in rice plants, such as rice growth stage, rice cultivars, and co-existing ions with Sb speciation in the environment. Another reason for this may be that Sb disrupts chlorophyll synthesis and damages the chloroplast structure.

Roots were more vulnerable to Sb toxicity. Sb(III) 20 mg L^−1^ uptake was greater than that of Sb(III) 10 mg L^−1^ in roots, while an non-significant change was observed in shoot Sb(V) accumulation (Figure 1c). The above results indicated that Sb(III) and Sb(V) both showed toxicity to rice plants, but the toxicity of Sb(III) was higher than Sb(V). Similar results were also observed earlier [46,49]. Roots accumulated more Sb than shoots of amaranth in amended soils, which was generally in line with our findings [9,50]. This might have deleterious effects since antimony accumulation in roots affects the distribution of nutrients and trace elements in plants. Nonetheless, shoot Sb(V) accumulation increased with the increase in the concentration of Sb [47]. In a fiber crop called *B. nivea*, its growth was significantly repressed with an increase in Sb levels [51]. Root and shoot Sb accumulation was negatively associated with rice plant vegetative growth (Table S2), which is indicative of the negative effects of Sb on rice. Sb toxicity can cause damage to plants phenotypically and physiologically, which negatively affects root and stem elongation and eventually plant growth.

Cellulose, hemicellulose, and pectin are the main polysaccharides in plant cell walls. Cell wall polysaccharides have been shown to contribute to the fixation of heavy metals [52]. High levels of Sb(III) and Sb(V) elevated uronic acid content in HCI and HCII but reduced pectin. HCII displayed a negative correlation with root and shoot Sb concentrations, root diameter, and XTH, while pectin showed a negative correlation with HCI (Table S2). Cellulose, pectin, and other components in the cell wall enclose a variety of negatively charged groups, which reduce metal ions employing complexation, adsorption, and precipitation [52]. Recently, it was concluded that hemicelluloses bind to cellulose and xyloglucans, mixed-linkage glucans, and xylans, and that their physical characteristics and interactions with cellulose result in the strengthening of the cell wall [21]. In a recent study, uronic acid contents of HCI and pectin in the root cell walls of *Athyrium wardii* increased significantly when they were exposed to Cd and Pb stress [10]. This suggests that uronic acid helps in enhancing the cell wall-binding capabilities of Sb. In previous studies, hemicellulose was found to be much more efficient in binding Cd^2+^ and Al^3+^ than pectin in root cell walls in the model plant *Arabidopsis thaliana*, thus being the prime pool for Cd [53] and Al accumulation in the roots of *A. thaliana* [54]. Pectins and pectinacious plant litters have been studied comprehensively as sources for the removal of heavy metal cations from wastewater [55]. Total sugar content of pectin and HCII was increased by Sb(III) (Figure 2d–f). Pectins and HCI are integral parts of cell wall components that are rich in sugars and uronic acids. They also play a crucial role in metal-binding cations through carboxyl groups [52,56,57]. Our results suggest this to be a cellular strategy to improve the metal chelation capacity in the rice cell wall matrix.

F1 (cell wall), F2 (organelle component), and F3 (cytosol) were screened for Sb accumulation (Figure 3). At higher concentrations of Sb(III), F1, F2, and F3 all displayed more Sb uptake. F1 and F2 had a maximum Sb(V) at low concentrations, while F3 showed a high accumulation of Sb(V) at higher concentrations. In recent studies, it was observed that Sb mainly accumulated in the cell wall and cytosol of *Phragmites australis* and *Potamogeton crispus* [58]. The cell wall and cytosol both play a role to safeguard plants from the harmful effects of Sb, as the cell wall structural components provide mechanical support and rigidity to plant [21].

We tried to obtain further insights into the toxicity of different forms of Sb on cell wall enzymes, e.g., XTH, CE (Figure 4), PG, PME, and β-gal (Figure 5). CE and PG enzymes exhibited a significant decrease at low Sb(III) 10 and Sb(V) 10 mg L^−1^. The toxicity of Sb was severe in PME and β-gal at higher concentrations of both forms of Sb. The activity of XTH was high in response to Sb(III) 20 and Sb(V) 10 mg L^−1^ (Figure 3). The reduced activity of cell wall-degrading enzymes, including PG), PME, and β-gal, in rice roots treated with Sb suggests a suppression of cell wall-loosening and -remodeling processes. Antimony has been shown to interact with cell wall enzyme catalytic sites, leading to enzyme inactivation or conformational changes that prevent catalytic efficiency [59]. The suppression of PG and PME activities may lead to pectin demethylation and depolymerization, which results in stiffer cell walls, which might restrict root elongation and nutrient transport [24,60]. Reduced β-galactosidase activity is able to affect cell expansion and signaling under stress [61,62]. The overall decrease in the enzymatic activities under Sb stress displays a defensive strategy of plants to cope with metal uptake by strengthening apoplastic obstruction, thereby reducing Sb mobility into the symplast [63]. Eventually, this synchronized suppression of cell wall-degrading enzymes emphasizes a strategic mechanical adaptation wherein rice roots intentionally stiffen their apoplastic matrix to establish a structural barrier that restricts antimony uptake and protects internal tissues.

PME displayed a positive correlation with root biomass, root surface area, and pectin content but a negative correlation with HCI. PG had a significantly negative correlation with HCI, while XTH showed a positive correlation with shoot root Sb. PMEs are notorious for being involved in plant growth and plant defense responses [28], as well as for increasing enzymatic activity under Cd stress in *Medicago sativa* stems [21]. XTHs are polysaccharides that have continued to attract much attention due to their crucial role in plant cell wall modeling [36]. Xyloglucan increased in response to Cd [21]. It was observed that both forms of Sb caused a negative effect on root cell wall enzymes. It is assumed that pectin in the cell walls of roots plays a role in constraining Sb outside the root cell of rice. The strong associations between these cell wall enzymes, hemicellulose (HCI) fractions, and pectin content confirm that plants dynamically restructure their cell wall polysaccharides to trap antimony, establishing a mechanical defense system that minimizes further toxic translocation.

The influence of different forms of Sb on morphological characteristics (root length, surface area, volume, average diameter, and fine root length) were investigated (Table S1). Root length, root surface area, and fine root length were reduced by higher concentrations of both speciation of Sb. Root biomass had a positive correlation with root length, root surface area, PME, and β-gal. Moreover, root length and root surface area displayed negative correlations with shoot Sb (Table S2). In recent studies, Sb(V) was accountable for a significant reduction in the surface area, volume the roots, and mean diameter, whereas Sb(III) reduced the most traits related to root morphology [49]. In another study, it was observed that the total root length and surface area decreased when the Sb(V) concentration was increased [47]. The negative effects of Sb on root morphological characteristics may be due to the deficient translocation of Sb from roots to shoots.

Finally, we tried to see the response of genes encoding root cell wall components to Sb(III) and Sb(V) using qRT-PCR for the determination of expression profiles of five randomly selected genes. Cell wall-associated proteins under Sb(III) and Sb(V) exposure revealed a strong link between Sb stress and cell wall-remodeling processes in rice roots. *XTH8* exhibited the most noticeable up-regulation, particularly under Sb(III) 20 treatment, suggesting that it has a major role in maintaining cell wall plasticity during metal stress. *XTHs* are known for mediating xyloglucan cleavage and rejoining, contributing to cell wall loosening and repair under abiotic stresses [64,65]. Similarly, *cellulose synthase* and *expansin OsEXPA1* exhibited moderate expression under Sb(III) and Sb(V), indicating that cell wall strength and extensibility are co-regulated as adaptive processes. *Expansins* facilitate cell wall loosening, improving root growth under stress, while *cellulose synthase* contributes to cell wall thickening to limit metal influx [66]. *Pectinesterase* expression was slightly enhanced under Sb(V) exposure, which may reflect its role in altering metal-binding capacity and modifying pectin cross-linkage to the cell wall [24]. In contrast, *xylanase* expression remained low across treatments, indicating its limited involvement in Sb stress responses or possible repression to preserve hemicellulose integrity. Generally, the up-regulation of these cell wall-linked genes exhibited the dynamic reassembling of rice root cell walls as a first-phase defense against Sb toxicity. This remodeling likely results in reduced Sb uptake, improved root mechanical stability, and sustained root growth under metalloid stress conditions. These diverse transcriptional profiles indicated that rice roots deploy a finely tuned molecular strategy, dynamically co-regulating cell wall loosening via *XTH8* and *OsEXPA1*, together with structural reinforcement through cellulose synthase, to restrict Sb influx and protect root mechanical stability. The differential expression of root-cell-wall-related genes perceived in this study is in line with previous transcriptomic evidence, showing that genes involved in cell wall biosynthesis and remodeling are responsive to arsenic and cadmium stress in rice roots [67]. Our results encompass these observations to Sb stress, signifying that Sb exposure may also prompt cell wall remodeling as part of the adaptive response.

Cis-regulatory elements in the promoter regions of rice root genes under antimony (Sb) stress showed a diverse set of hormone- and stress-responsive, conserved motifs, indicating complex transcriptional regulation (Table 1). The identification of ABA-responsive elements suggests that abscisic acid signaling plays a key role in the rice response to Sb-induced oxidative and osmotic stress. In previous research, it was reported that ABA enhances heavy metal tolerance through the modulation of ion transport and antioxidant defenses [68,69].

Similarly, the presence of DREs and LTREs implies that Sb stress induces cross-talk between cold, drought, and heavy metal signaling [70]. The detection MREs indicates specific transcriptional stimulation associated with metal detoxification processes; phytochelatin biosynthesis and metallothionein are known to mitigate antimony toxicity [71,72]. The G-box, a binding site for bZIP proteins and W-box, recognized by WRKY transcription factors, highlights the participation of both WRKY- and bZIP-mediated pathways in Sb stress responses, which are known to regulate redox balance, defense, and secondary metabolism upon metal exposure [73,74]. Moreover, the presence of MYB transcription factors contributes to the regulation of genes involved in oxidative stress tolerance and metal chelation [71,75]. HSE and ERE are critical components of stress adaptation in plants. These results indicate that Sb in root environment activates a wide transcriptional network, integrating oxidative and metal-responsive pathways to keep rice root homeostasis under metalloid stress.

Note: One limitation of our study is that the chemical speciation of Sb(III) and Sb(V) in the hydroponic solution were not directly monitored during and after the exposure period. Therefore, potential oxidation or reduction in Sb species cannot be completely excluded. Possible interconversion between Sb(III) and Sb(V) during hydroponic exposure cannot be ruled out. Consequently, our results should be interpreted based on the initially supplied Sb species.

## 4. Materials and Methods

The rice variety Yexiangyou 3 was used in this study. Selected uniform seeds were disinfected with 2% sodium hypochlorite (NaClO) solution for 10 min, followed by washing with de-ionized water. Next, they were sown on a moist culture medium (perlite: vermiculite = 1: 1; *v*/*v*) and watered regularly to ensure optimum moisture content for their germination. At the same time, 50% Arnon–Hoagland nutrient solution (50% HN nutrient solution, which comprised 2.5 mM KNO_3_, 0.5 mM NH4NO_3_, 0.5 mM NH_4_ H_2_PO_4_, 2 mM Ca(NO_3_)_2_·4H_2_O, 1 mM MgSO_4_·7H_2_O, 4.5 μM MnCl_2_·4H_2_O, 23 μM H_3_BO_3_, 0.4 μM, ZnSO_4_·7H_2_O, 0.15 μM CuSO_4_·5H_2_O, 0.05 μM H_2_MoO_4_, and 4.5 μMEDTA-Fe) was added dropwise to satisfy the nutritional needs of seedlings [76]. When the rice seedlings had two true leaves, ones with uniform growth were selected and transplanted into 50% Arnon–Hoagland nutrient solution for acclimation for 2 weeks. Later, nine seedlings were transferred to a 1 L capacity pot consisting of 50% Arnon–Hoagland nutrient solution (pH 5.5). After two weeks, different forms of Sb were added at the same time and placed in a greenhouse. The Sb used in the treatments was antimony potassium tartrate (KSbC_4_H_4_O_7_) and potassium pyroantimonate [K_2_H_2_Sb_2_O_7_·4H_2_O). The greenhouse conditions were as follows: a temperature of 25–30 °C, 100 μmol m^−2^ s^−1^ light intensity, a light/dark cycle of 16/8 h, and a relative humidity of 60–70 RH% [15]. Sampling was carried out after one week.

There were two parts to this experiment, and each experiment contained five treatments—control (CK, without Sb), 10 mg L^−1^ Sb(III), 20 mg L^−1^ Sb(III), 10 mg L^−1^ Sb(V), and 20 mg L^−1^ Sb(V)—with three replications each. During the acclimation period of seedlings, the nutrient solution was replaced every 3 days. The pH of the nutrient solution was adjusted to 5.5 with 0.1 mol L^−1^ NaOH and HNO_3_ [15].

1. To study the responses of root cell wall components, cell wall enzymes, and root morphology to different forms of Sb, the rice seedlings were harvested after two weeks exposure of different forms of Sb. The roots were separated from the seedlings and rinsed with distilled water, then immersed in a 20 mmolL^−1^ of ethylenediamine tetra acetic acid disodium dihydrate (C_10_H_14_N_2_Na_2_O_8_·2H_2_O) solution for 20 min. Collected samples were placed on filter paper to absorb the adherent moisture of the roots and above-ground rice seedlings, and their fresh weight was recorded. The parameters of root morphology were recorded using an Epson Scanner (Epson Expression 10000XL1.0, Shanghai Zhongjing Co., Ltd., Shanghai, China), and the data was analyzed using an equipped Root Image Analysis System accessed on 12 September 2024 (WinRHIZO 2020/2022). The data of the above parameters included root length, root volume, root surface area, root diameter, and the number of root tips. The aerial parts and the root samples were individually divided into two parts. One part was cut into small pieces, mixed, and oven-dried at 70 °C to a constant weight for 48 h to determine the total Sb concentration. For the other part, it was quickly frozen with liquid nitrogen and then stored in a −80 °C refrigerator to determine the contents of the cell wall components and cell wall enzymes.
2. To study the roles of root cell wall components in sequestrating different elements, the whole plant was washed with distilled water after exposure to above-mentioned treatments, followed by surface water drying with filter paper. The plants were divided into two parts, e.g., aerial part and roots. One part was cut into pieces, mixed, and dried in a 70 °C oven to a constant weight for 48 h. After digestion with HNO_3_, the samples were used to determine the total content of essential nutrients; the other part was rapidly frozen with liquid nitrogen and then stored in a refrigerator at −80 °C for the extraction of sub-cellular components and the determination of related elements.

Note: The experimental concentrations of Sb(III) and Sb(V) at 10 and 20 mg L^−1^ were selected based on environmentally realistic pollution baselines reported in severely degraded ecosystems. In areas surrounding active antimony mining in China, smelting, and technogenic waste centers, localized aqueous matrices and soil solutions often exhibit extreme Sb accumulations in this milligram-per-liter range. Thus, these concentrations represent severe, acute experimental stress conditions designed to deliberately challenge the crop’s physiological limits and elicit distinct morphological, physiological, and transcriptional responses in rice without causing immediate plant death. To prevent laboratory and environmental contamination, strict chemical disposal protocols were enforced post-harvest. All Sb-contaminated aqueous solutions and experimental pots were treated with hydrochloric acid HCl to neutralize and chemically stabilize the residual Sb(III) and Sb(V) complexes.

### 4.1. Extraction and Determination of Cell Wall-Related Enzymes

Approximately 0.15 g of fresh rice root samples was weighed and ground with a mortar to homogenate (1 g of tissue plus 9 mL of PBS pH 7.2–7.4). After that, the homogenate was transferred to a 2 mL centrifuge tube and centrifuged at 4000 rpm for 15 min. Then, the supernatant was collected and stored in a refrigerator at −20 °C until use [15]. The activities of PME, β-gal, CE, XTH, and PG in rice roots were determined using the ELISA kit (Shanghai Xinyu Biotechnology Co., Ltd., Shanghai, China) following the manufacturer’s protocol.

The idea was to cover the microporous plate with the pure enzyme antibody in order to create a solid phase antibody. An antibody–antigen enzyme-labeled antibody complex was generated by adding the enzyme to the microporous plate coated with the monoclonal antibody and joining it with the enzyme antibody labeled with horseradish peroxidase (HRP). Substrate 3,3′, 5,5′ tetramethyl-benzidine di-hydrochloride (TMB) was applied to develop color after thorough washing. HRP catalyzed the conversion of TMB to blue, and acid action changed it to its final yellow color. An iMark microplate reader (Beijing YuanYe BoLe Technology Development Co., Ltd., Beijing, China) was used to measure the absorbance (OD value) at a wavelength of 450 nm, and the activities of relevant enzymes in the cell wall of the plant roots were calculated according to a standard curve [77].

### 4.2. Extraction and Determination of Root Cell Wall Components

#### 4.2.1. Root Cell Wall Extraction

About 0.3 g of rice roots was weighed and placed in a pre-cooled mortar. Then, liquid nitrogen was added to grind the root samples into powder, which was then transferred to a 10 mL centrifuge tube. Furthermore, 75% pre-cooled ethanol (10 mL/g) was added to serve as an ice bath for 20 min. The mixed substances were then centrifuged at 10,000 rpm for 10 min at 4 °C, and then the supernatant was discarded. After that, the residues were extracted twice according to the above process. Subsequently, pre-cooled acetone (7 mL/g) was added into the centrifuge tube containing the residues, which was shaken, ice-bathed, and centrifuged at 4 °C for 20 min (centrifugation conditions were the same as above). After that, the supernatant was discarded, and a complex solution of chloroform/methanol (7 mL/g, 1:1 = *v*:*v*) was added. The complex solution was shaken well, and then the centrifugation was carried out after an ice bath for 20 min (the centrifugation conditions were the same as above). The supernatant was discarded again, and methanol (7 mL/g) was added. The centrifuge tubes were shaken and then centrifuged after an ice bath for 20 min (centrifugation conditions were the same as above). After discarding the supernatant, the precipitate was freeze-dried. The solid obtained after freeze-drying was the cell wall, which was stored at 4 °C until next use [78].

#### 4.2.2. Separation of Different Cell Wall Components

Pectin was extracted using a hot water bath method. Ammonium oxalate buffer solution containing 0.5% NaBH_4_ (pH = 4), 5 mg dried cell wall per 2 mL extraction solution, was added to the extracted cell wall. The above mixture was placed in a boiling water bath for 1 h, and it was then centrifuged at room temperature at 10,000 rpm for 10 min. The above process was repeated thrice, and the resulting supernatant was the pectin component. Next, 2 mL of 4% KOH was added to the precipitate, extracted for 12 h, and centrifuged at 10,000 rpm for 10 min at room temperature to collect the supernatant. The above steps were repeated 3 times, and the supernatant was collected as hemicelluloses I (HCI). Then, 1 mL of 24% KOH was added to the precipitate (5 mg dried cell wall per 1 mL extraction solution), extracted for 12 h, and centrifuged at 10,000 rpm for 10 min at room temperature. The above steps were repeated 3 times, and the resulting supernatant was hemicellulose II (HCII). The absorbance of the supernatant was also measured at 280 nm [79,80].

#### 4.2.3. Determination of Uronic Acid Content in Cell Wall Components

The first step for the determination of above parameters is to make a standard curve. We prepared the uronic acid standard solution (0.1 mg mL^−1^) and then pipetted 0, 0.2, 0.4, 0.6, 0.8, and 1 mL of the above standard solution into test tubes with stoppers. Then, the solution volume was topped up to 1 mL using distilled water and ice-bathed. At the same time, 10 mL of sodium tetraborate/sulfuric acid solution (0.95 g of Na_2_B_4_O_7_·10H_2_O in 100 mL of H_2_SO_4_) was added. After that, the mixture was blended with a vortex mixer and heated via a boiling water bath for 5 min. After cooling at room temperature, 0.1 mL of m-hydroxybiphenyl (0.15 g m-hydroxylbiphenyl) was dissolved in 100 mL of NaOH and fully mixed. The absorbance values for a 1 mL solution of the pectin component, along with 0.6 mL of HCI or HCII, were then measured following the same procedure outlined for the standard curve. The concentrations of each cell wall component were calculated based on the established standard curve [79,80,81].

### 4.3. Isolation of Sub-Cellular Components in Rice Roots

Differential centrifugation was performed to separate different cell components. Approximately 0.1 g of the root tissues was weighed and put in a mortar, and 10 mL of pre-cooled extraction solution (0.25 mmol·L^−1^ sucrose and 50 mmol·L^−1^ maleic acid salt, pH 7.5) was then added. Homogenized powdered root samples (50 mL) were centrifuged at 1000 g for 10 min at 4 °C. The residues in the centrifuge tubes were cell walls (F1). The supernatant was transferred into the other clean centrifuge tubes and then centrifuged at 12,000 rpm at 4 °C for 45 min. The residues comprised the organelle component (F2), and the supernatant was the cell cytosol component (F3).

The cell wall component (F1) and organelle component (F2) were oven-dried at 80 °C, and 10 mL of nitric acid was added for digestion. The concentrations of elements in the digested solution and the cytosol fraction (F3) were determined via inductively coupled plasma mass spectrometry (iCAP Qc ICP-MS, Thermo Fisher, Waltham, MA 02451, USA) [82,83].

### 4.4. Sample Digestion and Elemental Determination

Approximately 0.25 g of the dried and ground samples was weighed into a digestion tube with 10 mL of concentrated nitric acid kept overnight. An ED54 DigiBlock digestion system (Lab Tech, Inc., Hopkinton, MA, USA) was used for digestion [84]. The elemental content was determined by (ICP-MS) (iCAP Qc ICP-MS, Thermo Fisher, Waltham, MA 02451, USA). The reference material (bush leaves, GBW07603, GSV-2GBW10052) was processed synchronously with the samples to ensure the accuracy of the measurement. The reference material was obtained from the Center for Standard Reference of China and used to ensure the accuracy of the elemental analysis.

### 4.5. Measurement of Total Soluble Sugar Content

The shoots were dried and ground into fine powders with liquid nitrogen. The total soluble sugar was extracted and determined using the ‘Soluble Sugar Content Assay Kit’ (AKSU012C, Beijing Boxbio Science and Technology Co., Ltd., Beijing, China). Three independent biological replicates were sampled for this experiment [85].

### 4.6. Quantitative Real-Time PCR and Cis-Regulatory Elements Analysis

The extraction of total RNA from fully matured leaves of rice seedlings was performed using the TRIzol reagent according to the manufacturer’s protocols (Invitrogen, Waltham, MA 02451, USA); there were three replications carried out for each treatment, including Ck. The isolated RNA was treated with RNase-free DNase I (Promega, Tokyo, Japan). The final extracted RNA quality was assessed through NanoDrop (Thermo Fisher Scientific, Waltham, MA 02451, USA). Using 1.0–2.0 μg of RNA as templates (based on RNA concentration for each treatment), the Vazyme’s reverse transcriptase kit (HiScript^®^ Q Select RT SuperMix, Vazyme Biotech Co., Ltd., Nanjing, China) was utilized for reverse transcription and qRT-PCR analysis. The reverse transcription reaction was conducted at 25 °C for 10 min and then at 42 °C for 30 min, and it was finally stopped by heating at 85 °C for 5 min. The resulting cDNA was used as the template for real-time PCR amplification using gene-specific primers with NCBI Primer-BLAST (National Center for Biotechnology Information, Bethesda, MD, USA) (Supplementary Table S3) and SYBR green master mix. Candidate genes were selected based on prior studies, indicating their roles in rice root cell wall biosynthesis, remodeling, and expansion under heavy metal and metalloid stress [67]. Considering the resemblances between antimony and arsenic uptake pathways, we hypothesized that Sb exposure may also affect the expression of root-cell-wall-related genes. Therefore, *cellulose synthase*, *OsEXPA1*, *pectinesterase*, *XTH8*, and *xylanase* were selected for expression analysis. Gene sequences were acquired from the NCBI database and evaluated using the 2^−ΔΔCt^ method. The PCR conditions were as follows: 95 °C for 3 min followed by 30 cycles of 95 °C for 15 s, 58 °C 20 s, and 72 °C for 20 s. Three independent biological replicates were taken [86,87,88,89].

For *cis*-acting element analysis, we downloaded 1.5 Kb upstream of the *cellulose synthase-*, *expansin OsEXPA1-*, *pectinesterase-*, *XTH8-*, and *Xylanase*-encoding gene’s promoter sequences on Ensembl (http://plants.ensembl.org/index.html, accessed in 13 January 2025). Select ‘FASTA sequence’ format Output in ‘Export data’. The cis-acting elements predictions of the promoter were performed with PlantCare (http://bioinformatics.psb.ugent.be/webtools/plantcare/html/, accessed in 25 January 2025). After receiving data from PlantCare, the data was organized in table form [86].

To explore the correlations between morphological traits, physiological parameters, and root cell wall composition under (Sb(III) and (Sb(V)) stress, a Pearson’s correlation analysis was performed. Correlation coefficients (r) and their corresponding statistical significance (*p*-values) were calculated using GraphPad Prism 9. A *p*-value of < 0.05 was considered statistically significant [90].

### 4.7. Data Analysis

SPSS 20.0 software was used to analyze the variation between different treatments by using two-way analysis of variance (ANOVA) analysis. Prior to analysis, normality of residuals was calculated by employing the Shapiro–Wilk test, and homogeneity of variance was shown using Levene’s test, where the interaction was significant, simple effects were analyzed. Furthermore, a multiple comparison test (Tukey’s test) (*p* ≤ 0.05) was carried out. Multivariate variance analysis was carried out to assess the interaction between different levels of Sb (n = 3). Origin 9 software was used to draw the graphs.

## 5. Conclusions

The aim of the current study was to examine the toxicity of different forms of Sb (Sb(III) and Sb(V)) in rice plants (Yexiangyou 3) grown hydroponically. Sb is known to damage plant root, but there was scarce knowledge about Sb’s effects on root physiology, including root-cell-wall-related enzymes and genes. A higher concentration of Sb(III) adversely affected root morphological traits. Sb(III) and Sb(V) toxicity was greater in the cell organelle and cytosol. Activity of cellulose was distressed due to surplus Sb(III), while lower levels of Sb(V) affected PG contents. High concentrations of Sb(III) and Sb(V) caused significant decreases in PME and β-galactosidase. A high concentration of Sb(III) helped us to release a high amount of XTH. Pearson’s correlation analysis showed that shoot Sb had a positive correlation with XTH, root diameter, and HCI. Shoot biomass negatively affected root and shoot Sb, root diameter, XTH, and HCI. Pectin only had an antagonistic effect on HCI as it was a suitable biding site for uronic acid in Sb. HCI was negatively correlated with root morphological traits and pectin. To investigate the transcriptional regulatory mechanisms governing rice root cell wall modification under antimony (Sb) stress, an in silico analysis of the cis-acting regulatory elements was conducted. Root-related genes—*XTH8*, *pectinesterse*, *cellulase synthase* and *expansin*—were up-regulated in response to Sb. The cis-regulating elements in the promoter sequences of these genes had biotic and abiotic stress response elements, encompassing ABAs, MYBs, G-Box’s, DREs, metal response elements, and heat shock response elements, suggesting rice plant detoxification and defense mechanisms that limited Sb toxicity. Collectively, Sb species are responsible for the morphological and physiological changes in rice plant that appear after Sb contamination, while root-cell-wall-related genes can be considered as elements that can be utilized as remediation strategies for rice plant under Sb stress.

## Figures and Tables

**Table 1:** Predicted cis-regulatory elements of the promoter sequences of genes encoding rice root-related functions. Upstream promoter sequence (1.5 Kb) of five rice root-associated genes sequences that were downloaded from Ensembl (http://plants.ensembl.org/index.html, accessed on 13 January 2025) and were analyzed with PlantCARE (http://bioinformatics.psb.ugent.be/webtools/plantcare/html/, accessed on 25 January 2025).

Cis Elements	Consensus Sequence	Function
ABA-responsive element	ACGTG	Metalloids, drought, salt
Dehydration-responsive element (DRE)	RCCGAC	Drought, salt, cold (ABA-independent)
Low-temperature-responsive element (LTRE)	CCGAAA	Cold response
Anaerobic-responsive element (ARE)	TGGTTT	Low oxygen stress
Metal-responsive element (MRE)	TGCAGGC	Response to metals
W-box	TTGAC(C/T)	Pathogen response (WRKY binding)
G-box	CACGTG	Light, stress, ABA (bZIP TF binding)
MYB recognition sites	CAACTG or WAACCA	Drought, salt, cold, antioxidant defense
MYC recognition sites	CANNTG	Drought, salt
T/G-box	AACGTG	JA and pathogen response
HSE (heat shock element)	AGAAnnTTCT	Heat shock
ERE (ethylene-responsive element)	AWTTCAAA	Ethylene response

## Data Availability

Data are contained within this article and the Supplementary Materials.

## Supplementary Materials

The following supporting information can be downloaded at: https://www.mdpi.com/article/10.3390/plants15152297/s1, Table S1. Effects of different forms of antimony on the root length, surface area, volume, average diameter, tip number and fork number. Table S2. Pearson correlation analysis between different parameters studied in rice. Table S3. Primers used for qRT-PCR.

## Abbreviations

The following abbreviations are used in this manuscript:

Sb(III)	Antimonite
Sb(V)	Antimonate
PME	Pectin methylesterase
XTH	Xyloglucan endotransglycosylase hydrolase
PG	Polygalacturonase
HC-I,II	Hemicellulose-I,II
CE	Cellulase
