# Supplementary material for: Effect of Different Forms of Antimony (Sb) on Genes Encoding Functions Associated with Root Morphology, Physiology and Root Cell Wall in Rice (Oryza sativa)
Source: Plants (Basel). 2026 Jul 27;15(15):2297. doi: 10.3390/plants15152297 (PMC13468031; doi:10.3390/plants15152297)
Supplement: Supplementary file 1 [file plants-15-02297-s001.zip › plants-4413150-supplementary.pdf]

Supplementary Tables

Table S1. Effects of different forms of antimony on the root length, surface area, volume, average diameter, tip number and fork number.

Treatments ($mg\ L^{-1}$)	Length (cm)	Surface area (cm^2)	Volume (cm^3)	Average diameter (mm)	Tips	Forks
**CK**	436.0±10.04c[(1)]	45.17±1.65a	7.64±0.59a	0.96±0.03c	1785±10.50a	364.7±34.39c
**$Sb(III)_{10}$**	463.6±22.84a	49.33±2.28a	6.96±1.56ab	1.08±0.06ab	1209±38.16c	532.5±22.50a
**$Sb(III)_{20}$**	264.6±1.91b	31.36±3.64b	4.86±0.23c	1.11±0.02a	734.0±25.00d	312.5±14.50d
**$Sb(V)_{10}$**	463.7±23.96a	48.24±2.64a	5.22±0.65bc	0.97±0.05c	1545±60.67b	465.5±19.50b
**$Sb(V)_{20}$**	267.2±0.82b	27.21±0.69b	7.14±1.20a	1.00±0.04bc	1219±173.5c	335.5±10.50cd

(1) Different letters following values in the same column indicate significant differences ($p \leq 0.05$).

Table S2. Pearson correlation analysis between different parameters studied in rice

	Shoot biomass	Root biomass	Root Sb	Shoot Sb	Length	Area	Volume	Diameter	Tips	Forks	PME	β-gal	CE	PG	XTH	Pectin	HC1	HC2
Shoot biomass	1	0.475	-0.917**	-0.835**	0.182	0.046	0.389	-0.818**	0.782**	-0.148	-0.130	0.402	0.402	0.068	-0.634*	-0.221	-0.212	0.806**
Root biomass		1	-0.634*	-0.665**	0.647**	0.587*	0.458	-0.535*	0.748**	0.156	0.650**	0.526*	0.300	0.266	-0.508	0.419	-0.778**	0.435
Root Sb			1	0.959**	-0.419	-0.272	-0.568*	0.813**	-0.894**	-0.109	-0.059	-0.284	-0.192	-0.291	0.846**	0.188	0.352	-0.869**
Shoot Sb				1	-0.595*	-0.476	-0.531*	0.790**	-0.902**	-0.324	-0.103	-0.343	-0.124	-0.187	0.826**	0.224	0.327	-0.775**
Length					1	0.971**	0.160	-0.334	0.680**	0.801**	0.483	0.528*	0.026	0.086	-0.403	0.078	-0.430	0.207
Area						1	0.070	-0.210	0.567*	0.813**	0.520*	0.532*	-0.073	0.006	-0.271	0.167	-0.430	0.040
Volume							1	-0.237	0.374	0.024	0.175	-0.205	-0.230	0.460	-0.753**	-0.063	-0.311	0.706**
Diameter								1	-0.758**	-0.030	-0.050	-0.432	-0.442	-0.045	0.464	0.095	0.256	-0.726**
Tips									1	0.286	0.354	0.569*	0.264	0.319	-0.677**	0.089	-0.593*	0.685**
Forks										1	0.094	0.110	-0.397	-0.020	-0.295	-0.272	0.046	-0.013
PME											1	0.365	0.087	0.455	-0.003	0.804**	-0.886**	-0.053
β-gal												1	0.650**	-0.249	0.129	0.382	-0.462	0.114
CE													1	-0.212	0.234	0.191	-0.160	0.189
PG														1	-0.444	0.334	-0.557*	0.325
XTH															1	0.340	0.222	-0.760**
Pectin																1	-0.809**	-0.239
HC1																	1	-0.192
HC2																		1

** $P < 0.01$

* $P < 0.05$

Table S3. Primers used for qRT-PCR.

S.no	Genes	Primer Sequence 5′- 3′
1	cellulose synthase-like protein E1 F	TCTACCCTCACCACACTCTTAC
	cellulose synthase-like protein E1 R	CACACGCAGAGCACTTACTATC
2	Expansin OsEXPA1 F	GGAACGATGGGTAAGTTCGTAG
	Expansin OsEXPA1 R	TATAGCTCGCCACACCAAAC
3	Pectinesterase Rice F	GGCTGAAAGGAGGAGCAATA
	Pectinesterase Rice R	CTAGTAGATCGAGTCGCCAAAC
4	XTH 8 F	TCCACCACCCAAAGACAAAG
	XTH 8 R	GTAGAACTGGTGCTCCTTCTTG
5	Xylanase F	AGTCGAGGGCTACACCTATTA
	Xylanase R	CTCTGTCTGTCAATCGCCTATC
6	Actin F	TCCATCTTGGCATCTCTCAG
	Actin R	GTACCCGCATCAGGCATCTG
